# *N*-(2-Fluoro­benzo­yl)-*N*′,*N*′′-bis­(4-methyl­phen­yl)phospho­ric triamide

**DOI:** 10.1107/S1600536811009640

**Published:** 2011-03-19

**Authors:** Mehrdad Pourayoubi, Atekeh Tarahhomi, Arnold L. Rheingold, James A. Golen

**Affiliations:** a Department of Chemistry, Ferdowsi University of Mashhad, Mashhad 91779, Iran; b Department of Chemistry, University of California, San Diego, 9500 Gilman Drive, La Jolla, CA 92093, USA

## Abstract

The P atom in the title compound, C_21_H_21_FN_3_O_2_P, is in a tetra­hedral coordination environment and the environment of each N atom is essentially planar (sums of angles = 359.7, 359.9 and 358.4°). The phosphoryl and carbonyl groups adopt *anti* orientations with respect to each other. In the crystal, adjacent mol­ecules are linked *via* N—H⋯O=P and two N—H⋯O=C hydrogen bonds into an extended chain parallel to the *a* axis.

## Related literature

For a phospho­rus ligand having a C(O)NHP(O) skeleton, see: Gholivand *et al.* (2010 ▶). For a related structure, see: Pourayoubi *et al.* (2010 ▶). For bond lengths in related structures, see: Sabbaghi *et al.* (2010 ▶) and references cited therein.
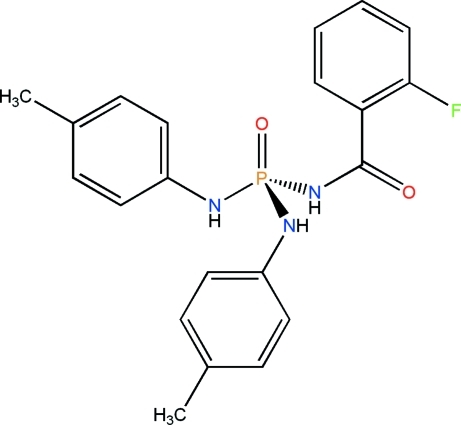


## Experimental

### 

#### Crystal data

C_21_H_21_FN_3_O_2_P
*M* *_r_* = 397.38
Monoclinic, 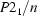

*a* = 9.7697 (9) Å
*b* = 10.2197 (9) Å
*c* = 20.2404 (18) Å
β = 96.605 (1)°
*V* = 2007.5 (3) Å^3^
*Z* = 4
Mo *K*α radiation
μ = 0.17 mm^−1^
*T* = 100 K
0.25 × 0.15 × 0.15 mm

#### Data collection

Bruker SMART CCD area-detector diffractometer
Absorption correction: multi-scan (*SADABS*; Bruker, 2005 ▶) *T*_min_ = 0.959, *T*_max_ = 0.975
15865 measured reflections
4551 independent reflections
3411 reflections with *I* > 2σ(*I*)
*R*_int_ = 0.035

#### Refinement

*R*[*F* ^2^ > 2σ(*F* ^2^)] = 0.046
*wR*(*F* ^2^) = 0.127
*S* = 1.05
4551 reflections
264 parameters
3 restraints
H atoms treated by a mixture of independent and constrained refinement
Δρ_max_ = 0.41 e Å^−3^
Δρ_min_ = −0.26 e Å^−3^

### 

Data collection: *SMART* (Bruker, 2005 ▶); cell refinement: *SAINT* (Bruker, 2005 ▶); data reduction: *SAINT*; program(s) used to solve structure: *SHELXS97* (Sheldrick, 2008 ▶); program(s) used to refine structure: *SHELXL97* (Sheldrick, 2008 ▶); molecular graphics: *SHELXTL* (Sheldrick, 2008 ▶); software used to prepare material for publication: *SHELXTL* and *enCIFer* (Allen *et al.*, 2004 ▶).

## Supplementary Material

Crystal structure: contains datablocks I, global. DOI: 10.1107/S1600536811009640/nc2222sup1.cif

Structure factors: contains datablocks I. DOI: 10.1107/S1600536811009640/nc2222Isup2.hkl

Additional supplementary materials:  crystallographic information; 3D view; checkCIF report

## Figures and Tables

**Table 1:** Hydrogen-bond geometry (Å, °)

*D*—H⋯*A*	*D*—H	H⋯*A*	*D*⋯*A*	*D*—H⋯*A*
N1—H1*N*⋯O2^i^	0.87 (1)	1.92 (1)	2.780 (2)	171 (2)
N2—H2*N*⋯O1^ii^	0.86 (1)	2.08 (1)	2.886 (2)	156 (2)
N3—H3*N*⋯O1^ii^	0.86 (1)	2.24 (2)	2.945 (2)	139 (2)

Symmetry codes: (i) 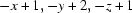; (ii) 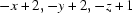.

**Fig. 1.**
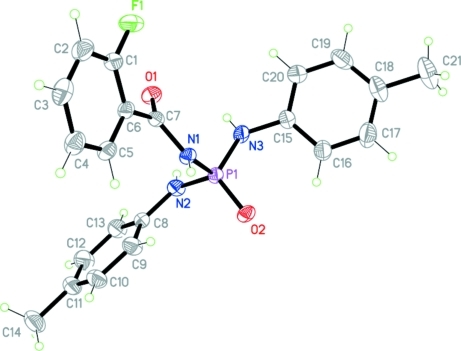
An ORTEP-style plot of title compound with labeling. Displacement ellipsoids are drawn at the 50% probability level.

## supplementary crystallographic information

### Comment

Carbacylamidophosphates with a C(O)NHP(O) skeleton have attracted attention because of their roles as the *O*,*O'*-donor ligands for metal complexation (Gholivand *et al.*, 2010). Following the previous works about carbacylamidophosphates such as P(O)[NHC(O)C_6_H_3_(2,6-F_2_)][N(CH_3_)(CH_2_C_6_H_5_)]_2_ (Pourayoubi *et al.*, 2010), here, we report on the synthesis and crystal structure of the title compound, P(O)[NHC(O)C_6_H_4_(2-F)][NH—C_6_H_4_-4-CH_3_]_2_.

In the crystal structure of the title compound the phosphoryl and carbonyl groups adopt *anti* positions to each other. The P atom has a slightly distorted tetrahedral configuration (Fig. 1). The bond angles around the P atom are in the range of 101.09 (8)° to 116.67 (8)°. The P1—N2 and P1—N3 bonds (1.6361 (15) Å and 1.6291 (17) Å) are shorter than the P1—N1 bond (1.6872 (15) Å). The environment of the nitrogen atoms is essentially planar. The P═O bond length of 1.4723 (13) Å is comparable to those in similar compounds *e.g.* in P(O)[NHC(O)C_6_H_4_(4-NO_2_)][NHC_6_H_11_]_2_ (Sabbaghi *et al.*, 2010).

In the crystal structure, adjacent molecules are linked *via* N_C(O)NHP(O)_—H···O ═P and two N_amide_—H···O ═C hydrogen bonds (see Table 1), into an extended chain parallel to the *a* axis.

### Experimental

2-F—C_6_H_4_C(O)NHP(O)Cl_2_ has been synthesized from the reaction between phosphorus pentachloride (4.0 g, 19.2 mmol) and 2-fluorobenzamide (2.671 g, 19.2 mmol) in dry CCl_4_ at 358 K (3 h) and then the treatment of formic acid (0.884 g, 19.2 mmol) at ice bath temperature. To a solution of 2-F—C_6_H_4_C(O)NHP(O)Cl_2_ (0.3 g, 1.17 mmol) in dry chloroform (30 ml), a mixture of *p*-toluidine (0.251 g, 2.34 mmol) and triethylamine (0.237 g, 2.34 mmol) in dry chloroform (10 ml) was added at 273 k. After 4 h stirring, the solvent was removed and the product was washed with distilled water and recrystallized from methanol/chloroform at room temperature. IR (KBr, cm^-1^): 3308 (NH), 3030 (NH), 2896, 2627, 1639 (C═O), 1457, 1220, 1061, 944, 795.

### Refinement

Hydrogen atoms H1N, H2N, and H3N were located in Fourier difference map and were refined with *DFIX* 0.88 (0.01) for the N–H bond lengths and isotropic displacement parameter of 1.2 times *U*_eq_ of the parent N atoms. All other hydrogen atoms were placed in their calculated positions with atom–H lengths of 0.95 Å (CH) and 0.98 Å (CH3) and isotropic displacement parameters for these atoms were set to 1.20 times (CH) and 1.50 times (CH_3_) *U*_eq_ of the parent C atom.

### Crystal data

**Table:**

C_21_H_21_FN_3_O_2_P	*F*(000) = 832
*M**_r_* = 397.38	*D*_x_ = 1.315 Mg m^−^^3^
Monoclinic, *P*2_1_/*n*	Mo *K*α radiation, λ = 0.71073 Å
*a* = 9.7697 (9) Å	Cell parameters from 5744 reflections
*b* = 10.2197 (9) Å	θ = 2.2–27.5°
*c* = 20.2404 (18) Å	µ = 0.17 mm^−^^1^
β = 96.605 (1)°	*T* = 100 K
*V* = 2007.5 (3) Å^3^	Block, colourless
*Z* = 4	0.25 × 0.15 × 0.15 mm

### Data collection

**Table:**

Bruker SMART CCD area-detector diffractometer	4551 independent reflections
Radiation source: fine-focus sealed tube	3411 reflections with *I* > 2σ(*I*)
graphite	*R*_int_ = 0.035
φ and ω scans	θ_max_ = 27.9°, θ_min_ = 2.0°
Absorption correction: multi-scan (*SADABS*; Bruker, 2005)	*h* = −12→12
*T*_min_ = 0.959, *T*_max_ = 0.975	*k* = −11→13
15865 measured reflections	*l* = −25→25

### Refinement

**Table:**

Refinement on *F*^2^	Primary atom site location: structure-invariant direct methods
Least-squares matrix: full	Secondary atom site location: difference Fourier map
*R*[*F*^2^ > 2σ(*F*^2^)] = 0.046	Hydrogen site location: inferred from neighbouring sites
*wR*(*F*^2^) = 0.127	H atoms treated by a mixture of independent and constrained refinement
*S* = 1.05	*w* = 1/[σ^2^(*F*_o_^2^) + (0.0669*P*)^2^ + 0.498*P*] where *P* = (*F*_o_^2^ + 2*F*_c_^2^)/3
4551 reflections	(Δ/σ)_max_ = 0.007
264 parameters	Δρ_max_ = 0.41 e Å^−^^3^
3 restraints	Δρ_min_ = −0.26 e Å^−^^3^

### Special details

**Table:**

Geometry. All e.s.d.'s (except the e.s.d. in the dihedral angle between two l.s. planes) are estimated using the full covariance matrix. The cell e.s.d.'s are taken into account individually in the estimation of e.s.d.'s in distances, angles and torsion angles; correlations between e.s.d.'s in cell parameters are only used when they are defined by crystal symmetry. An approximate (isotropic) treatment of cell e.s.d.'s is used for estimating e.s.d.'s involving l.s. planes.
Refinement. Refinement of *F*^2^ against ALL reflections. The weighted *R*-factor *wR* and goodness of fit *S* are based on *F*^2^, conventional *R*-factors *R* are based on *F*, with *F* set to zero for negative *F*^2^. The threshold expression of *F*^2^ > σ(*F*^2^) is used only for calculating *R*-factors(gt) *etc*. and is not relevant to the choice of reflections for refinement. *R*-factors based on *F*^2^ are statistically about twice as large as those based on *F*, and *R*- factors based on ALL data will be even larger.

### Fractional atomic coordinates and isotropic or equivalent isotropic displacement parameters (Å^2^)

**Table:**

	*x*	*y*	*z*	*U*_iso_*/*U*_eq_	
P1	0.72255 (5)	0.92119 (4)	0.50855 (2)	0.02136 (14)	
F1	0.92737 (14)	1.40039 (12)	0.55287 (6)	0.0441 (3)	
O1	0.91560 (13)	1.14443 (13)	0.50431 (6)	0.0293 (3)	
O2	0.58757 (12)	0.86140 (12)	0.51487 (6)	0.0250 (3)	
N1	0.69300 (16)	1.08365 (14)	0.50258 (7)	0.0210 (3)	
H1N	0.6074 (11)	1.1095 (19)	0.4983 (9)	0.025*	
N2	0.79772 (16)	0.87866 (15)	0.44363 (8)	0.0240 (3)	
H2N	0.8811 (12)	0.8507 (19)	0.4525 (10)	0.029*	
N3	0.84260 (16)	0.88686 (16)	0.56874 (8)	0.0267 (4)	
H3N	0.9270 (12)	0.905 (2)	0.5636 (10)	0.032*	
C1	0.8139 (2)	1.41838 (19)	0.50898 (9)	0.0296 (4)	
C2	0.7701 (2)	1.5451 (2)	0.49516 (11)	0.0381 (5)	
H2C	0.8194	1.6173	0.5157	0.046*	
C3	0.6533 (2)	1.5647 (2)	0.45090 (11)	0.0379 (5)	
H3B	0.6219	1.6511	0.4408	0.045*	
C4	0.5822 (2)	1.4596 (2)	0.42132 (11)	0.0344 (5)	
H4A	0.5029	1.4737	0.3903	0.041*	
C5	0.6265 (2)	1.33331 (19)	0.43680 (9)	0.0278 (4)	
H5A	0.5765	1.2612	0.4166	0.033*	
C6	0.74372 (19)	1.31052 (17)	0.48164 (9)	0.0230 (4)	
C7	0.79305 (18)	1.17460 (18)	0.49728 (8)	0.0220 (4)	
C8	0.7441 (2)	0.89104 (17)	0.37612 (9)	0.0244 (4)	
C9	0.6203 (2)	0.9545 (2)	0.35614 (10)	0.0319 (5)	
H9A	0.5685	0.9914	0.3884	0.038*	
C10	0.5725 (2)	0.9640 (2)	0.28921 (10)	0.0360 (5)	
H10A	0.4886	1.0089	0.2762	0.043*	
C11	0.6439 (2)	0.9096 (2)	0.24070 (10)	0.0404 (5)	
C12	0.7657 (2)	0.8443 (2)	0.26118 (10)	0.0419 (6)	
H12A	0.8157	0.8051	0.2288	0.050*	
C13	0.8164 (2)	0.8347 (2)	0.32801 (10)	0.0341 (5)	
H13A	0.9004	0.7898	0.3409	0.041*	
C14	0.5884 (3)	0.9202 (3)	0.16774 (11)	0.0561 (7)	
H14A	0.6098	0.8398	0.1446	0.084*	
H14B	0.6313	0.9950	0.1479	0.084*	
H14C	0.4884	0.9326	0.1636	0.084*	
C15	0.8269 (2)	0.85706 (18)	0.63606 (9)	0.0260 (4)	
C16	0.7217 (2)	0.7756 (2)	0.65213 (10)	0.0406 (5)	
H16A	0.6570	0.7408	0.6180	0.049*	
C17	0.7115 (2)	0.7452 (3)	0.71817 (11)	0.0506 (7)	
H17A	0.6367	0.6929	0.7288	0.061*	
C18	0.8072 (3)	0.7891 (2)	0.76906 (10)	0.0435 (6)	
C19	0.9162 (3)	0.8633 (2)	0.75138 (11)	0.0507 (7)	
H19A	0.9862	0.8909	0.7850	0.061*	
C20	0.9253 (3)	0.8980 (2)	0.68584 (11)	0.0427 (6)	
H20A	1.0000	0.9504	0.6752	0.051*	
C21	0.7964 (3)	0.7510 (3)	0.84055 (11)	0.0657 (8)	
H21A	0.8791	0.7801	0.8687	0.099*	
H21B	0.7879	0.6557	0.8437	0.099*	
H21C	0.7150	0.7926	0.8556	0.099*	

### Atomic displacement parameters (Å^2^)

**Table:**

	*U*^11^	*U*^22^	*U*^33^	*U*^12^	*U*^13^	*U*^23^
P1	0.0143 (3)	0.0263 (2)	0.0236 (2)	0.00103 (18)	0.00229 (18)	0.00314 (18)
F1	0.0442 (8)	0.0440 (7)	0.0404 (7)	−0.0137 (6)	−0.0116 (6)	−0.0023 (5)
O1	0.0150 (7)	0.0379 (8)	0.0351 (8)	−0.0004 (6)	0.0030 (6)	0.0005 (6)
O2	0.0142 (7)	0.0278 (7)	0.0330 (7)	−0.0001 (5)	0.0025 (5)	0.0038 (5)
N1	0.0143 (8)	0.0244 (8)	0.0245 (8)	0.0002 (6)	0.0038 (6)	0.0007 (6)
N2	0.0158 (8)	0.0297 (8)	0.0262 (8)	0.0048 (6)	0.0015 (6)	−0.0003 (6)
N3	0.0133 (8)	0.0388 (9)	0.0279 (9)	0.0000 (7)	0.0018 (7)	0.0086 (6)
C1	0.0288 (11)	0.0346 (11)	0.0252 (10)	−0.0079 (9)	0.0024 (8)	−0.0004 (8)
C2	0.0497 (15)	0.0292 (10)	0.0368 (12)	−0.0104 (10)	0.0106 (10)	−0.0051 (9)
C3	0.0433 (14)	0.0280 (11)	0.0443 (12)	0.0005 (9)	0.0135 (11)	0.0062 (9)
C4	0.0271 (11)	0.0353 (11)	0.0411 (12)	0.0002 (9)	0.0058 (9)	0.0084 (9)
C5	0.0231 (10)	0.0292 (10)	0.0317 (10)	−0.0033 (8)	0.0057 (8)	0.0011 (8)
C6	0.0202 (10)	0.0273 (9)	0.0227 (9)	−0.0035 (7)	0.0073 (7)	−0.0015 (7)
C7	0.0167 (10)	0.0303 (9)	0.0192 (9)	−0.0013 (7)	0.0033 (7)	−0.0019 (7)
C8	0.0212 (10)	0.0274 (9)	0.0247 (9)	−0.0034 (8)	0.0032 (8)	0.0010 (7)
C9	0.0270 (11)	0.0404 (11)	0.0278 (10)	0.0020 (9)	0.0019 (8)	−0.0030 (8)
C10	0.0290 (12)	0.0461 (12)	0.0313 (11)	0.0001 (10)	−0.0038 (9)	0.0029 (9)
C11	0.0345 (13)	0.0601 (15)	0.0262 (11)	−0.0110 (11)	0.0015 (9)	0.0024 (9)
C12	0.0351 (13)	0.0624 (15)	0.0300 (12)	−0.0047 (11)	0.0109 (10)	−0.0062 (10)
C13	0.0254 (11)	0.0452 (12)	0.0324 (11)	0.0013 (9)	0.0060 (9)	−0.0017 (9)
C14	0.0465 (16)	0.095 (2)	0.0262 (12)	−0.0108 (14)	0.0008 (11)	0.0069 (12)
C15	0.0234 (10)	0.0299 (10)	0.0248 (10)	0.0057 (8)	0.0040 (8)	0.0025 (7)
C16	0.0266 (12)	0.0627 (15)	0.0318 (11)	−0.0063 (10)	0.0001 (9)	0.0129 (10)
C17	0.0322 (13)	0.0782 (18)	0.0421 (14)	−0.0019 (12)	0.0075 (11)	0.0229 (12)
C18	0.0496 (15)	0.0558 (14)	0.0263 (11)	0.0136 (12)	0.0098 (10)	0.0039 (9)
C19	0.0709 (19)	0.0504 (14)	0.0284 (12)	−0.0100 (13)	−0.0048 (12)	−0.0038 (10)
C20	0.0499 (15)	0.0433 (13)	0.0336 (12)	−0.0166 (11)	−0.0006 (10)	0.0005 (9)
C21	0.074 (2)	0.095 (2)	0.0310 (13)	0.0190 (17)	0.0163 (13)	0.0111 (13)

### Geometric parameters (Å, °)

**Table:**

P1—O2	1.4723 (13)	C9—H9A	0.9500
P1—N3	1.6291 (17)	C10—C11	1.385 (3)
P1—N2	1.6361 (15)	C10—H10A	0.9500
P1—N1	1.6872 (15)	C11—C12	1.386 (3)
F1—C1	1.351 (2)	C11—C14	1.517 (3)
O1—C7	1.229 (2)	C12—C13	1.389 (3)
N1—C7	1.362 (2)	C12—H12A	0.9500
N1—H1N	0.872 (9)	C13—H13A	0.9500
N2—C8	1.412 (2)	C14—H14A	0.9800
N2—H2N	0.862 (9)	C14—H14B	0.9800
N3—C15	1.422 (2)	C14—H14C	0.9800
N3—H3N	0.863 (9)	C15—C20	1.375 (3)
C1—C6	1.380 (3)	C15—C16	1.390 (3)
C1—C2	1.382 (3)	C16—C17	1.387 (3)
C2—C3	1.382 (3)	C16—H16A	0.9500
C2—H2C	0.9500	C17—C18	1.384 (3)
C3—C4	1.378 (3)	C17—H17A	0.9500
C3—H3B	0.9500	C18—C19	1.387 (3)
C4—C5	1.385 (3)	C18—C21	1.514 (3)
C4—H4A	0.9500	C19—C20	1.386 (3)
C5—C6	1.396 (3)	C19—H19A	0.9500
C5—H5A	0.9500	C20—H20A	0.9500
C6—C7	1.492 (3)	C21—H21A	0.9800
C8—C9	1.391 (3)	C21—H21B	0.9800
C8—C13	1.392 (3)	C21—H21C	0.9800
C9—C10	1.384 (3)		
			
O2—P1—N3	114.88 (8)	C9—C10—C11	121.7 (2)
O2—P1—N2	116.67 (8)	C9—C10—H10A	119.2
N3—P1—N2	101.09 (8)	C11—C10—H10A	119.2
O2—P1—N1	105.49 (8)	C10—C11—C12	117.8 (2)
N3—P1—N1	111.59 (8)	C10—C11—C14	120.6 (2)
N2—P1—N1	107.03 (8)	C12—C11—C14	121.6 (2)
C7—N1—P1	123.97 (13)	C11—C12—C13	121.5 (2)
C7—N1—H1N	118.3 (13)	C11—C12—H12A	119.2
P1—N1—H1N	117.4 (13)	C13—C12—H12A	119.2
C8—N2—P1	127.06 (13)	C12—C13—C8	120.0 (2)
C8—N2—H2N	117.9 (13)	C12—C13—H13A	120.0
P1—N2—H2N	114.9 (13)	C8—C13—H13A	120.0
C15—N3—P1	127.97 (13)	C11—C14—H14A	109.5
C15—N3—H3N	111.7 (14)	C11—C14—H14B	109.5
P1—N3—H3N	118.7 (14)	H14A—C14—H14B	109.5
F1—C1—C6	119.15 (18)	C11—C14—H14C	109.5
F1—C1—C2	118.14 (18)	H14A—C14—H14C	109.5
C6—C1—C2	122.7 (2)	H14B—C14—H14C	109.5
C3—C2—C1	118.7 (2)	C20—C15—C16	118.94 (18)
C3—C2—H2C	120.7	C20—C15—N3	119.60 (18)
C1—C2—H2C	120.7	C16—C15—N3	121.11 (18)
C4—C3—C2	120.4 (2)	C17—C16—C15	119.8 (2)
C4—C3—H3B	119.8	C17—C16—H16A	120.1
C2—C3—H3B	119.8	C15—C16—H16A	120.1
C3—C4—C5	119.9 (2)	C18—C17—C16	121.8 (2)
C3—C4—H4A	120.0	C18—C17—H17A	119.1
C5—C4—H4A	120.0	C16—C17—H17A	119.1
C4—C5—C6	120.93 (19)	C17—C18—C19	117.3 (2)
C4—C5—H5A	119.5	C17—C18—C21	120.8 (2)
C6—C5—H5A	119.5	C19—C18—C21	121.8 (2)
C1—C6—C5	117.37 (18)	C20—C19—C18	121.5 (2)
C1—C6—C7	121.68 (18)	C20—C19—H19A	119.2
C5—C6—C7	120.92 (16)	C18—C19—H19A	119.2
O1—C7—N1	121.22 (17)	C15—C20—C19	120.5 (2)
O1—C7—C6	123.01 (16)	C15—C20—H20A	119.8
N1—C7—C6	115.77 (15)	C19—C20—H20A	119.8
C9—C8—C13	118.93 (18)	C18—C21—H21A	109.5
C9—C8—N2	122.47 (16)	C18—C21—H21B	109.5
C13—C8—N2	118.58 (17)	H21A—C21—H21B	109.5
C10—C9—C8	120.08 (18)	C18—C21—H21C	109.5
C10—C9—H9A	120.0	H21A—C21—H21C	109.5
C8—C9—H9A	120.0	H21B—C21—H21C	109.5
			
O2—P1—N1—C7	178.66 (14)	C5—C6—C7—N1	−39.0 (2)
N3—P1—N1—C7	53.27 (16)	P1—N2—C8—C9	6.1 (3)
N2—P1—N1—C7	−56.46 (16)	P1—N2—C8—C13	−172.26 (15)
O2—P1—N2—C8	57.27 (18)	C13—C8—C9—C10	−1.7 (3)
N3—P1—N2—C8	−177.42 (15)	N2—C8—C9—C10	179.93 (18)
N1—P1—N2—C8	−60.53 (17)	C8—C9—C10—C11	1.1 (3)
O2—P1—N3—C15	−28.7 (2)	C9—C10—C11—C12	0.3 (3)
N2—P1—N3—C15	−155.21 (16)	C9—C10—C11—C14	179.3 (2)
N1—P1—N3—C15	91.30 (17)	C10—C11—C12—C13	−0.9 (3)
F1—C1—C2—C3	−179.31 (17)	C14—C11—C12—C13	180.0 (2)
C6—C1—C2—C3	−1.7 (3)	C11—C12—C13—C8	0.3 (3)
C1—C2—C3—C4	0.1 (3)	C9—C8—C13—C12	1.0 (3)
C2—C3—C4—C5	1.1 (3)	N2—C8—C13—C12	179.47 (19)
C3—C4—C5—C6	−0.7 (3)	P1—N3—C15—C20	−144.91 (18)
F1—C1—C6—C5	179.64 (15)	P1—N3—C15—C16	41.9 (3)
C2—C1—C6—C5	2.0 (3)	C20—C15—C16—C17	4.9 (3)
F1—C1—C6—C7	−2.4 (3)	N3—C15—C16—C17	178.1 (2)
C2—C1—C6—C7	179.95 (17)	C15—C16—C17—C18	−3.0 (4)
C4—C5—C6—C1	−0.8 (3)	C16—C17—C18—C19	−1.2 (4)
C4—C5—C6—C7	−178.77 (17)	C16—C17—C18—C21	−178.0 (2)
P1—N1—C7—O1	−8.6 (2)	C17—C18—C19—C20	3.3 (4)
P1—N1—C7—C6	170.55 (12)	C21—C18—C19—C20	−179.9 (2)
C1—C6—C7—O1	−37.7 (3)	C16—C15—C20—C19	−2.8 (3)
C5—C6—C7—O1	140.17 (18)	N3—C15—C20—C19	−176.1 (2)
C1—C6—C7—N1	143.13 (17)	C18—C19—C20—C15	−1.4 (4)

### Hydrogen-bond geometry (Å, °)

**Table:**

*D*—H···*A*	*D*—H	H···*A*	*D*···*A*	*D*—H···*A*
N1—H1N···O2^i^	0.87 (1)	1.92 (1)	2.780 (2)	171 (2)
N2—H2N···O1^ii^	0.86 (1)	2.08 (1)	2.886 (2)	156 (2)
N3—H3N···O1^ii^	0.86 (1)	2.24 (2)	2.945 (2)	139 (2)

Symmetry codes: (i) −*x*+1, −*y*+2, −*z*+1; (ii) −*x*+2, −*y*+2, −*z*+1.
